# Immune checkpoint inhibitors in resectable non-small cell lung cancer: recent successes and ongoing challenges

**DOI:** 10.3389/fonc.2026.1779081

**Published:** 2026-03-31

**Authors:** Faith O. Abodunrin, Robert B. Cameron, Waqas Haque, Christine M. Bestvina, Marina C. Garassino

**Affiliations:** Section of Hematology/Oncology, Department of Medicine, University of Chicago, Chicago, IL, United States

**Keywords:** adjuvant, biomarker, immunotherapy, lung cancer, neoadjuvant, perioperative

## Abstract

Systemic treatment for resectable early-stage non-small cell lung cancer (NSCLC) has historically consisted of chemotherapy, with limited efficacy and substantial toxicity. Immune checkpoint inhibitors (ICI) have found increasing use in neoadjuvant, adjuvant, and perioperative settings. In this review, we summarize major clinical trials using ICI in the adjuvant, neoadjuvant, and perioperative settings. We further discuss novel biomarkers, including pathologic response, ctDNA, genomic and protein expression patterns, and machine learning approaches such as radiomics and digital pathology. Finally, we discuss recent and ongoing trials of different escalation approaches.

## 1 Introduction

The recognition, control, and elimination of tumors by the immune system are key innate anti-tumor responses. (1). To evade these responses, tumors can increase their expression of programmed death ligand-1 (PD-L1), which provides an inhibitory signal to T cells by binding to programmed cell death-1 receptor (PD-1). Immune checkpoint inhibitors (ICI) overcome this immunosuppression by blocking inhibitory proteins on tumor cells and T cells, thereby re-engaging the antitumor immune response. As T cells recognize tumor neoantigens, antitumor immunity develops and ideally provides a durable response against tumors.

Historical five-year survival for patients with stage II and IIIA non-small cell lung cancer following tumor resection is 40% and 25%, respectively. (2). Perioperative chemotherapy has been explored as a strategy to reduce the risk for disease recurrence and improve overall survival (OS). This strategy, however, offers only a 5% improvement in 5-year survival rate compared to surgery alone (3, 4). Thus, seeking alternative modalities to improve outcome in these NSCLC (71) patients has been an important topic of research. Because ICI had success in metastatic NSCLC, trials over the last decade have examined the role of ICI following surgical resection (adjuvant), prior to surgical resection (neoadjuvant), and prior to and following surgical resection (perioperative). In this review, we outline the biological and clinical rationale for these approaches; existing and investigational biomarkers to predict patient outcomes; and ongoing trials of novel strategies to improve outcomes for early-stage NSCLC.

## 2 Clinical trials in early-stage NSCLC

### 2.1 Adjuvant ICI in NSCLC

The goal of adjuvant immunotherapy after surgical resection is to eradicate persistent micro metastatic disease by overcoming the postoperative immunosuppressed state (5). Surgical removal of the primary tumor and nodal disease can increase the production of inflammatory cytokines such as IL-6, IL-8, IL-10, and TNF. This postoperative proinflammatory state results in the expansion of immunosuppressive cell subsets including regulatory T-cells, myeloid-derived suppressor cells, and tumor associated macrophages. Such immunosuppression in the tumor microenvironment and systemically in the immediate postoperative phase can potentially be reversed by immune checkpoint inhibitors (6). With the lower postsurgical neoplastic burden, adjuvant immunotherapy can thus lead to effective eradication of minimal residual disease (7).

This rationale has been tested in several randomized clinical trials. PEARLS/KEYNOTE-091 was a randomized, international, phase 3 trial investigating adjuvant pembrolizumab in patients with completely resected stage IB-IIIA NSCLC (8). The primary dual endpoints were disease-free survival (DFS) in the overall population and in the PD-L1 TPS ≥ 50% population. Following surgery, patients were randomized to receive adjuvant pembrolizumab or placebo every 3 weeks for up to 18 cycles. In the overall population, 3-year DFS was 58% in the pembrolizumab arm compared to 50% in the placebo arm (HR 0.76, 0.63-0.91). At the time of the prespecified analysis, the median DFS and median OS had not been reached in both placebo and pembrolizumab arms. Similarly, the IMpower-010 trial examined the role of atezolizumab following adjuvant platinum-based chemotherapy in patients with surgically resected stage IB-IIIA NSCLC with a primary endpoint of DFS. In this study, adjuvant atezolizumab improved DFS with a trend towards improved OS in patients with stage II-IIIA disease with PD-L1expression >1% (9–11). Within this population, the 5-year DFS of 53.2% vs 42.7% for best-supportive care (HR 0.70, 0.55-0.91) and 5-years OS 74.8% vs. 66.3% (HR 0.77, 0.56-1.06). The benefit was specifically pronounced in patients with PD-L1 expression ≥ 50% with 5-years OS 82.7% vs 65.3% (HR 0.47, 0.28-0.77) (11). However, in the ITT population, there were no statistically significant differences in DFS or OS. These two studies demonstrated the potential utility of adjuvant immunotherapy for patients with resected NSCLC, but their modest effect size led to the consideration of alternative strategies for implementing immunotherapy in the treatment of early-stage NSCLC. **Table 1** includes select adjuvant immune checkpoint inhibitor trials in lung cancer.

**Table 1:** Selected trials of adjuvant immunotherapy.

Clinical trial (NCT number)	Study population	Intervention	mDFS (HR, 95% CI)	mOS (HR, 95% CI)
IMPOWER010 (NCT02486718)	Resected stage II-IIIA following adjuvant chemotherapy	Adjuvant atezolizumab vs. best supportive care	ITT: 65.6 mo vs. 47.8 mo (0.85, 0.71-1.01) PD-L1≥1%: 68.5 mo vs. 37.3 mo (0.70, 0.55-0.91)	ITT: NE vs. NE (0.97, 0.78-1.22) PD-L1≥1%: NE vs. 87.1 mo (0.77, 0.56-1.06)
KEYNOTE -091/PEARLS (NCT02504372)	Resected stage IB-IIIA following adjuvant chemotherapy	Adjuvant pembrolizumab vs. placebo	53.6 mo vs. 42.0 mo (0.76, 0.63-0.91)	NE vs. NE (0.87, 0.67-1.15)

### 2.2 Neoadjuvant ICI in NSCLC

Neoadjuvant immunotherapy is postulated to be an effective strategy given the larger neoantigen repertoire and low clonal heterogeneity prior to the removal of the primary tumor. Theoretically, the high antigen burden results in a robust T cell response and thus ensures optimization of immunotherapy efficacy (7). Additionally, neoadjuvant ICI use induces a stable T cell response with tumor-specific memory T cell expansion which translates to durable clinical benefit (12). Furthermore, when activated, T cells are mobilized via the lymphatic and bloodstream to the primary tumor and nodal sites. The lymphatic and blood circulatory system is intact prior to surgery and thus local delivery of T cells is uninhibited (13). A study by Liu et al. using murine models of breast cancer determined that the efficacy of anti-PD-1 and anti-CTLA-4 in the neoadjuvant setting was better when compared to the use in the adjuvant setting. The neoadjuvant approach resulted in superior eradication of micro metastases and improved survival (14). The study also showed that higher number of tumor infiltrating cells is associated with improved prognosis.

One of the earliest clinical studies to investigate the role of neoadjuvant immunotherapy in NSCLC was CheckMate 159 (15). This phase Ib/II study included 21 untreated patients with stage I-IIIA disease who received two cycles of the anti-PD-1 monoclonal antibody nivolumab preoperatively. Most patients (20 out of 21) underwent surgical resection, and the regimen did not lead to surgical delay. The rate of major pathological response (MPR, which is defined as ≤ 10% residual viable tumor cells in the surgical specimen) was 45%, with the rate of pathologic complete response rate (pCR, defined as no residual viable tumor cells in either the primary tumor or nodal site) of 10%. Updated analysis of this study showed that the 5-year recurrence free survival and OS were 60% and 80%, respectively. Among those who achieved MPR, 89% had not experienced death or recurrence at 5 years (16). These outcomes, particularly with regard to pathologic responses, were substantially improved compared to historical studies of neoadjuvant chemotherapy and prompted further studies incorporating neoadjuvant immunotherapy for NSCLC. Several subsequent early phase studies have investigated incorporating either single agent anti-PD-1 or anti-PD-L1 or in combination with anti-CTLA4 antibodies or other novel immune targeting antibodies such as anti-LAG3, anti-STAT3, anti-NKG2A, and others (17–20).

In the single-arm phase II LCMC study (18), 181 stage IB-IIIB NSCLC with resectable disease received two doses of neoadjuvant atezolizumab monotherapy. Despite including a high-risk population with approximately 50% of all patients having clinical stage III disease, 159 out of 181 patients (88%) underwent surgery. Out of the 159 patients who underwent surgery, about 10% of the tumors harbored either EGFR mutations (exon 19 deletion, n=8; exon 20 insertion, n-1; L858R, n=1) or ALK rearrangements (n=6). The primary end point for the study was MPR, and this was 20% (29 out of 143, 95% CI, 14-28%) in the primary efficacy analysis excluding those with activating mutations. Of note, those with EGFR or ALK alterations did not achieve MPR or show radiographical response. In the phase 2 NEOSTAR study, neoadjuvant nivolumab and nivolumab plus ipilimumab were investigated in 44 operable NSCLC patients. The dual checkpoint inhibitor arm resulted in MPR rates of 50% (8/16) while the single agent nivolumab arm achieved 24% (5/21) (20).

The first approved neoadjuvant-only chemoimmunotherapy regimen was based on results from CheckMate 816. In this international phase III study, patients with resectable stage IB-IIIA NSCLC were randomized to receive three cycles of neoadjuvant nivolumab plus platinum-based chemotherapy or platinum-based chemotherapy (21). Like other neoadjuvant ICI studies, the addition of neoadjuvant nivolumab did not result in surgical delay, as 83% of the patients in the nivolumab arm completed definite surgery compared to 75% in the chemotherapy arm. The study demonstrated a significant improvement in the dual primary endpoints: pCR rates were 24% versus 2.2%, and median event-free survival (EFS) of 31.6 months compared to 20.8 months (HR 0.63, 0.43 to 0.9). The incidence of grade 3–4 treatment related adverse events (TRAE) were comparable in both arms (33.6% versus 36.9% in the chemotherapy arm). Additionally, in an updated report, 65.4% of patients who received neoadjuvant nivolumab and chemotherapy were alive compared to 55% (95% CI, 47.3-62.0) in the chemotherapy only group at five years (22). Given the surgical feasibility, tolerability, and efficacy of this regimen, additional trials investigated the potential of combining neoadjuvant chemoimmunotherapy with adjuvant immunotherapy as a perioperative treatment strategy. **Table 2** includes select neoadjuvant immunotherapy studies in lung cancer.

**Table 2:** Selected trials of neoadjuvant immunotherapy.

Clinical trial (NCT number)	Study population	Intervention	Pathologic responses	Surgical rates	mEFS (HR, 95% CI)	mOS (HR, 95% CI)
CHECKMATE 159 (NCT02259621)	Resectable stage I-IIIA	Nivolumab administered 28 and 14 days prior to surgery	MPR: 45% pCR: 10%	20/21 patients	Median not reached; 5-year RFS 60%	Median not reached; 5 year OS 80%
NEOSTAR (NCT03158129)	Stage IA-IIIA	Arm A: Nivolumab 3mg/kg x3 doses prior to surgery Arm B: Nivolumab 3mg/kg and ipilimumab 1mg/kg x3 doses prior to surgery	Arm A: MPR 22% pCR 9% Arm B: MPR 38% pCR 29%	Arm A: 95.6% Arm B: 81.0%	Arm A: NE; 24 month RFS 82.6% Arm B: NE; 24-month RFS 81.0%	Arm A: NE; 24-month OS 96% Arm B: NE; 24-month OS 95.2%
CHECKMATE 816 (NCT02998528)	Resectable stage IB (≥ 4cm)- IIIA NSCLC	Platinum doublet chemotherapy+ nivolumab vs. placebo for 3 cycles prior to surgery	MPR:36.9% vs. 8.9% pCR 24.0% vs. 2.2%	83.2% vs 75.4%	31.6 mo vs. 20.8 mo (0.63, 0.43-0.91)	Median not reached; 5-year OS 65.4% vs. 55.0% (0.72, 0.523-0.998)
NeoCOAST (NCT03794544)	Resectable stage I(>2 cm) to IIIA	Durvalumab+ chemotherapy+: Arm 1: Oleclumab Arm 2: Monalizumab Arm 4: Datopotamab deruxtecan	Arm 1: MPR 41.9%, pCR 20.3% Arm 2: MPR 50.0%, pCR 25.7% Arm 4: MPR: 63.0%, pCR 35.2%	Arm 1: 93.2% Arm 2: 93.0% Arm 4: 94.4%	Not reported	Not reported

### 2.3 Perioperative ICI in NSCLC

KEYNOTE-671 was the first international, randomized phase III study investigating the addition of perioperative immunotherapy (neoadjuvant and adjuvant) to chemotherapy to reduce the risk for disease recurrence in patients with resectable NSCLC (23). In the study, 797 patients with resectable stage II-IIIB NSCLC either received four cycles of neoadjuvant platinum-based chemotherapy with pembrolizumab followed by surgery and adjuvant pembrolizumab for up to 13 cycles or neoadjuvant platinum-based chemotherapy plus placebo followed by surgery and adjuvant placebo. This study used OS and EFS as dual primary endpoints, making it the only perioperative trial investigating ICIs that used OS as an endpoint. Surgical resection was completed in 82.1% of the patients in the pembrolizumab group compared to 79.4% in the chemotherapy only group. At 24 months, the EFS was 62.4% in the pembrolizumab arm compared to 40.5% in the placebo arm (HR:0.58, 95% CI: 0.46-0.72). MPR rates tripled in the pembrolizumab group compared to the placebo group (30.2% vs 11.0%) while pCR rates more than quadrupled (18.1% vs 4%). Although no statistically significant difference was seen in the first interim analysis, updated second interim analysis of the study showed 36-month overall survival estimates of 71% (95% CI 66-76) in the pembrolizumab group and 64% in the placebo group (HR 0.72, 0.56-0.93). This study also allowed participants harboring EGFR or ALK gene alterations and these patients were found to have lower EFS compared to wild-type participants (24).

The use of perioperative durvalumab in resectable NSCLC was investigated in the international, phase 3, placebo-controlled, AEGEAN trial (25). 802 patients with stage IIA-IIIB NSCLC were randomized to receive either 4 cycles of platinum-based chemotherapy with a fixed dose of durvalumab or placebo every 3 weeks. Following surgery, patients continued to receive either placebo or durvalumab every 4 weeks for up to 12 cycles. The primary endpoints of the study were event free survival (EFS) and pCR. EFS in the modified intention-to treat population which excluded patients with EGFR and ALK alterations was significantly longer in the durvalumab group than in the placebo group. The EFS at 12 months was 73.4% for patients in the durvalumab group and 64.5% for the patients in the placebo group. There was also a pCR advantage noted in the durvalumab group with pCR rate of 17.2% compared to 4.3% in the placebo group.

Another perioperative study conducted in China was the Neotorch trial which investigated the combination of platinum-based chemotherapy and toripalimab (PD-1 inhibitor) or placebo for 3 cycles (26). After surgery, patients received an additional cycle of toripalimab or placebo with platinum-based chemotherapy and then followed by either toripalimab or placebo every 3 weeks for up to 13 cycles. The co-primary endpoints were EFS and MPR. At the prespecified interim analysis, the median EFS was not estimable in the toripalimab arm compared to 15.1 months in the placebo arm (HR, 0.40, 0.28-0.57). A 40-percentage point difference in major pathological response rate was seen between both groups (48.5% in the toripalimab arm vs. 8.4% in the control group). PD-L1 status was predictive of improved outcomes as participants with PD-L1 levels ≥ 1% had numerically improved EFS outcomes (HR 0.65, 0.33-1.23 in PD-L1expression <1% group; HR 0.31, 0.17-0.54 in PD-L1expression 1%-49%). The incidence of grade 3 or 4 TRAEs were 63.4% in the toripalimab groups vs 54% in the control group.

The CheckMate 77T study randomized patients with stage IIA-IIIB resectable NSCLC to perioperative nivolumab plus chemotherapy or perioperative placebo plus chemotherapy (27). This study used EFS as a primary endpoint with pCR, MPR, and OS as secondary endpoints. At prespecified interim analysis, the 18-month EFS was 70.2% in the nivolumab arm compared to 50.0% in the placebo arm (HR 0.56, 0.41-0.76) with pCR rates of 25.3% vs 4.7% and MPR rates of 35.4% vs 12.1%. The incidence of grade 3 or 4 TRAEs were 25.2% in the chemotherapy arm compared to 32.5% in the nivolumab arm; notably, in the adjuvant period 8.5% of patients developed grade 3 or 4 TRAE with nivolumab compared to 2.6% with placebo.

Together, these studies of perioperative immune checkpoint inhibition have demonstrated the feasibility, safety, and efficacy of this approach in patients with resectable NSCLC. However, the increase in grade 3 or 4 TRAEs, particularly in the adjuvant setting, raises the question of whether all patients benefit from the adjuvant phase of immunotherapy. For instance, these studies have unsurprisingly demonstrated that patients harboring alterations in EGFR or ALK receive no benefit from immunotherapy (consistent with findings in metastatic disease). Identifying other biomarkers that predict improved or poorer patient outcomes can help tailor treatment plans for patients to avoid over- or under-treatment. See **Table 3** for select perioperative clinical trials in lung cancer.

**Table 3:** Selected trials of perioperative immunotherapy.

Clinical trial (NCT number)	Study population	Intervention	Pathologic responses	Surgical rates	mEFS (HR, 95% CI)	mOS (HR, 95% CI)
NADIM (NCT03081689)	Stage IIIA	Nivolumab+ carboplatin+paclitaxel q3w for 3 cycles; adjuvant nivolumab for 1 year	MPR 82.9%, pCR 63.4%	89.1%	NE; 36-month PFS 69.6%	NE; 36-month OS 81.9%
LCMC3 (NCT02927301)	Stage IB-IIIB	Atezolizumab 1200 mg, Q3w 2 cycles; optional adjuvant atezolizumab for 1 year	MPR 20% pCR 6%	88%	NE; 36-month DFS 72%	NE; 36-month OS 80%
AEGEAN (NCT03800134)	Stage IIA-IIIB	Durvalumab vs. placebo + chemotherapy for 4 cycles before surgery, adjuvant durvalumab vs. placebo x12 cycles after surgery	MPR: 33.3% vs 12.3% pCR: 17.2% vs 4.3%	77.6% vs 76.7%	NE vs. 25.9 mo (0.68, 0.53-0.88)	NE vs 53.2 mo (0.89, 0.70-1.14)
CHECKMATE 77T (NCT04025879)	Stage IB-IIIB	Nivolumab vs placebo +chemotherapy for 4 cycles; adjuvant nivolumab x 12 cycles	MPR 35.4% vs. 12.1% pCR: 25.3% vs. 4.7%	77.7% vs 76.7%	46.6 vs. 16.9 mo (0.61, 0.46-0.80)	NE vs. NE (0.85, 0.58-1.25), 30-month OS 78% vs 72%
KEYNOTE 671 (NCT03425643)	Stage IIB-IIIA	Pembrolizumab vs. placebo + chemotherapy x 4 cycles; adjuvant pembrolizumab vs. placebo q3w for 13 cycles	MPR 30.2% vs 11.0% pCR 18.1% vs. 4.0%	79.4% vs. 66.9%	47.2 mo vs 18.3 mo (0.59, 0.48-0.72)	NE vs. 52.4 mo (0.72, 0.56-0.93)
NEOTORCH (NCT04158440)	Stage II-III NSCLC	Toripalimab vs. placebo + platinum-based chemo for 3 cycles; adjuvant toripalimab vs. placebo+ chemotherapy x1 cycle followed by toripalimab vs. placebo x 13 cycles	MPR 48.5% vs 8.3% pCR 24.8% vs 1.0%	82.2% vs. 73.3%	NE vs. 15.1 mo (0.40, 0.28-0.57)	NE vs. 30.4 mo (0.62, 0.38-1.00)
RATIONALE-315 (NCT04379635)	Stage II-IIIA	Tislelizumab vs. placebo+ chemotherapy x 3–4 cycles; adjuvant tislelizumab vs. placebo q6w x 8 cycles	MPR: 56% vs. 15% pCR: 41% vs. 6%	84.1% vs. 76.2%	NE vs. NE (0.56, 0.40-0.79), 24-month EFS 68% vs. 52%	NE vs. NE (0.62, 0.39-0.98); 24-month OS 89% vs. 79%

## 3 Biomarkers for more personalized treatments

As patients undergo care for early-stage NSCLC, there are multiple opportunities for biomarker discovery. Between biopsies, surgical resections, imaging, and laboratory testing, there are rich opportunities to identify features that associate with patient outcomes. Here, we outline several established and investigational biomarkers that can guide patient care with early-stage NSCLC.

### 3.1 Pathologic responses

Surgical resection of tumors represents a unique opportunity to assess the effect of treatment at the cellular level. Pathologists measure the residual viable tumor (RVT) at the time of surgical resection to identify the proportion of the tumor that was killed by treatment (28). This value can be further stratified as a pCR (RVT of 0%) MPR (RVT 0-10%) (29). These values have been used as surrogate endpoints for survival in other tumor types, such as breast and bladder cancer following neoadjuvant chemotherapy (30, 31). Based on these results in other tumor types, there has been consideration of using pathologic responses as surrogate endpoints in NSCLC.

Within the relatively nascent space of neoadjuvant therapy for NSCLC, MPR and pCR have frequently been used as primary or co-primary endpoints to obtain more expedient results regarding therapeutic efficacy. Perhaps unsurprisingly, neoadjuvant chemoimmunotherapy increases the rate of pCR and MPR compared to chemotherapy alone, and patients who achieve a pCR or an MPR have improved survival compared to those who do not (21–23, 27). These results have raised the possibility that pathologic responses might represent a surrogate endpoint for either EFS or OS. A meta-analysis to address this question demonstrated that pathologic responses correlate well with indexed survival, emphasizing the prognostic importance of these endpoints (32). However, trial-level surrogacy (that is, the correlation between the OR of a pathologic response with the HR for survival endpoints across different studies) was moderate and imprecise. As such, pathologic responses alone are not an adequate surrogate for regulatory decisions for neoadjuvant or perioperative chemoimmunotherapy regimens.

Nonetheless, a patient’s pathologic response provides key prognostic information to clinicians. By evaluating RVT at surgery, pathologists and oncologists can infer the systemic efficacy of treatment. In the case of a pCR, patients have no RVT in the primary tumor or sampled lymph nodes, indicating eradication of macroscopic disease. Given the low rates of pCR with chemotherapy alone, ICI is contributing to the response and may have established effective and durable immune memory. This is borne out in trials that report survival stratified by pathologic response, with excellent and durable EFS and OS regardless of treatment arm as seen in CheckMate 816, CheckMate 77T, and KEYNOTE 671 and suggests that those patients who achieve a pCR with neoadjuvant chemoimmunotherapy are effectively cured and can transition to surveillance following surgery.

In contrast, patients who achieve a MPR but not a pCR have low levels of RVT in the primary tumor. This incomplete treatment effect raises the possibility of some persistent micrometastatic disease, which, if left unchecked, might recur. This hypothesis is borne out by the 5-year EFS and OS from CheckMate 816 for patients achieving a MPR being numerically lower than those who achieved a pCR. In this study, patients achieving a MPR had 5-year EFS rates of 75% and 61% in the nivolumab and placebo arms, respectively, while patients with a pCR had EFS rates of 88% and 100%, respectively. Notably, the EFS rates at 12 months were similar between MPR (92% vs. 94% for nivolumab vs. placebo) and pCR (95% vs. 100% for nivolumab vs. placebo). Furthermore, the patients achieving an MPR from nivolumab had numerically higher 5-year EFS compared to those receiving chemotherapy alone. This finding may suggest a degree of durable immune control in these patients that might be augmented by additional ICI. As the 12- and 24-month EFS were similar in patients with a MPR compared to those with a pCR in CheckMate 77T and KEYNOTE 671, longer follow up is needed to better delineate the survival benefit of adjuvant immunotherapy in patients achieving an MPR. Pending these results, implementing adjuvant ICI is an attractive option for patients achieving an MPR but not a pCR.

Finally, patients who achieve neither a pCR nor an MPR, EFS and OS are substantially worse. Survival data from CheckMate 816 show that patients who achieve neither an MPR nor a pCR have 5-year EFS rates of 32% in the nivolumab arm and 31% in the placebo arm and 5-year OS rates of 52.8% and 52.3%. These similar EFS and OS rates indicate that patients with RVT>10% require additional treatment. Indeed, results from CheckMate 77T and KEYNOTE 671 show higher EFS rates in such patients who receive perioperative immunotherapy compared to neoadjuvant chemotherapy. The lack of granularity in reported RVT complicates this decision, however. A patient with a RVT of 15% clearly has a better treatment response than a patient with an RVT of 80%, and the optimal adjuvant therapy for two such patients likely should differ. As discussed below, many biomarkers are under investigation to better identify which patients are more likely to benefit from neoadjuvant, adjuvant, or perioperative ICI, and novel therapeutic strategies may help appropriately escalate therapies for patients with suboptimal responses.

### 3.2 Clinical and genomic biomarkers

Advances in treating metastatic NSCLC have been guided by the identification of biomarkers to better personalize therapy. Among patients without actionable driver mutations, high PD-L1 expression has been used to identify patients for whom chemotherapy might be omitted in favor of ICI monotherapy (33, 34). In contrast, for patients with mutations in STK11 or KEAP1, treatment with PD-L1 inhibitors have been associated with worse survival (35), suggesting that these genomic alterations are associated with resistance to immunotherapy. However, these associations were identified in the metastatic setting, where systemic therapy is a staple of therapy for most patients. In the early-stage setting, surgical resection is the major driver of longer survival, and the importance of these biomarkers may be lessened.

In neoadjuvant and perioperative trials, subgroup analyses have shown benefit across PD-L1 expression, albeit with numerically lower rates of pCR and shorter EFS in patients with PD-L1 expression <1%. In the adjuvant setting, the PEARLS/KEYNOTE-091 study showed similar disease-free survival across PD-L1 expression with HR 0.78, 0.67, and 0.82 for patients with PD-L1 expression <1%, 1-49%, and >50%, respectively. (8) Conversely, the IMpower-010 study showed worse DFS in patients with PD-L1 <1% (HR 0.97 vs. 0.66 for patients with PD-L1 expression >1%). (9) A meta-analysis of early-stage NSCLC trials found no interaction effect of PD-L1 expression with pathologic responses in the neoadjuvant setting. (36) While there was a significant interaction effect between PD-L1 expression and EFS in neoadjuvant and perioperative studies, there was no interaction between PD-L1 expression status and DFS in the adjuvant setting. This meta-analysis reiterated the favorable effect of chemoimmunotherapy in the neoadjuvant and adjuvant setting for patients with PD-L1 negative tumors. Furthermore, real-world data demonstrate a weak correlation between PD-L1 expression and %RVT in surgical resection specimens (37), and 40% of patients with PD-L1 negative tumors achieved a pCR in this study. Thus, while higher PD-L1 expression may be associated with deeper pathologic responses, other factors play a stronger role in driving outcomes for patients with early-stage NSCLC. Currently, for patients with early-stage NSCLC with PD-L1 expression <1%, the appropriate treatment regimen is chemoimmunotherapy, with the timing being guided by the patient’s stage at presentation. In such patients who require adjuvant treatment (e.g., those with negative nodal staging on EBUS but positive nodes on surgery), pembrolizumab is the appropriate immunotherapy given the consistent benefit across PD-L1 expression levels. Since patients with PD-L1 expression<1% receive a numerically smaller benefit from chemoimmunotherapy, improving outcomes in this population represents an opportunity for the development of novel trials to better personalize care based on PD-L1 expression status.

The role of genomic alterations in driving neoadjuvant immunotherapy responses likewise remains understudied. KEAP1 and STK11 have established importance in the metastatic setting as negative predictors of ICI response (38–40), and in the early-stage setting, mutations in STK11 are associated with worse pathologic response rates and even early disease progression (41–43). This is likely due to the distinct immune profiles observed in such tumors, with lower PD-L1 expression and transcriptional evidence of T-cell dysfunction. In the metastatic setting, CTLA4 inhibition can overcome this immunosuppressive microenvironment (44, 45), but in the neoadjuvant setting, dual checkpoint inhibition is a less proven strategy. Exploratory analyses of the ipilimumab+nivolumab arm from CheckMate 816 showed numerically improved EFS, OS, and pathologic responses compared to chemotherapy (46). Despite these efficacy signals, more patients in the ipilimumab+nivolumab arm were unable to undergo surgical resection. These reports did not perform additional analyses to address how many patients in this arm had tumors with STK11 or KEAP1 alterations. As such, the role of dual checkpoint inhibition in the early-stage setting for these patients remains unclear, with potential safety concerns competing with rationale for enhanced efficacy.

### 3.3 Circulating tumor DNA

Circulating tumor DNA (ctDNA) can be detected in the bloodstream for the identification of tumor-derived mutations. Assays to detect ctDNA can be tumor agnostic (i.e., detecting a panel of specific mutations, regardless of their presence in a tumor) or tumor informed (i.e., detecting specific genomic alterations identified from sequencing a tumor sample) (47, 48). The former has established utility in NSCLC to accelerate the identification of actionable genomic alterations such as EGFR with high specificity. On the other hand, the latter has better sensitivity, and efforts have been made to utilize tumor-informed approaches for the identification of minimal residual disease in NSCLC. The detection of ctDNA after definitive surgery correlates with the risk of recurrence across multiple studies (48, 49), and those with negative ctDNA testing have a lower likelihood of recurrence (50). A recent meta-analysis comparing tumor-informed and tumor-agnostic approaches found that longitudinal monitoring led to similar sensitivity of the two approaches for disease recurrence, though tumor-informed assays had higher specificity (51).

In trials of neoadjuvant chemoimmunotherapy in NSCLC, patients with initially positive ctDNA who clear their ctDNA have improved EFS and OS compared to those who have persistently positive ctDNA after the start of neoadjuvant therapy (22, 52, 53). However, in contrast to pathologic response, immunotherapy tends to improve outcomes compared to chemotherapy in patients with negative ctDNA testing (22, 52), and not all patients with ctDNA clearance achieve pCR. These findings suggest that ctDNA clearance does not necessarily indicate a disease-free state and that more sensitive testing is needed for ctDNA clearance alone to be used to de-escalate therapy following neoadjuvant chemoimmunotherapy.

As yet, while persistent ctDNA positivity represents a negative prognostic factor, there are not yet results to guide treatment in patients who do not clear their ctDNA after neoadjuvant treatment. Prior observational studies have shown improved outcomes with adjuvant therapy in those who have positive ctDNA testing after surgery (54, 55), but these patients had not received neoadjuvant therapy and received adjuvant chemotherapy or tyrosine kinase inhibitor rather than immunotherapy. For now, ctDNA positivity and ctDNA clearance can provide prognostic information for clinicians, but there are currently insufficient data to make decisions on therapy escalation or de-escalation based on ctDNA alone.

### 3.4 Machine learning approaches: radiomics and digital pathology

Patients with NSCLC undergo imaging with CT or PET imaging as part of their initial staging and to follow their treatment response. The acquisition of multiple images through a patient’s course provides a high content set of data. However, radiographic responses alone can correlate poorly with pathologic responses. Leveraging machine learning approaches to identify potentially relevant radiographic features generates models that can be used to predict patient outcomes, including pathologic responses or survival. Numerous retrospective studies have used radiomics to generate such models, and these often outperform models based solely on clinical features. While these studies have been retrospective and therefore may have less utility for general clinic practice, they have provided some insights in terms of model generation for the use of radiomics in NSCLC.

First, radiomic models benefit from the integration of complementary datasets. Early studies in patients undergoing neoadjuvant chemoradiation generated models using solely radiographic features such as lymph node homogeneity and tumor sphericity (56). Subsequent studies have integrated other imaging modalities such as PET (57); clinical and pathologic features (58, 59); and pre- and post-treatment imaging (58, 60), and these integrated models have had higher AUC. However, even with these integrated models, the AUC of these models have largely hovered between 0.7-0.8. Additional models with larger sample sizes might help overcome these limitations, and additional prospective validation is needed to determine how radiomics might fit into standard clinical practice.

Perhaps the more tantalizing outcome of these radiomic studies has been its ability to infer tumor biology. Several studies have provided biological validation of their radiomic models using gene expression data (57, 61). These studies have identified transcriptional programs, including proliferation and immune cell infiltration, that are associated with greater predicted pathologic response. By correlating the output of radiomic models with biologically relevant information, these models can provide preliminary data for hypothesis generation, such as how genomic alterations or transcriptional profiles might impact baseline tumor morphology or even how these genomic and transcriptomic features might associate with therapeutic response or resistance.

In addition to leveraging artificial intelligence to develop radiomic models, other groups have used computational approaches with pathology specimens to identify prognostic features for patients with early-stage NSCLC. Some groups have used digital pathology approaches to identify novel prognostic biomarkers on tissue samples. One group used tissue microarray with immunohistochemistry to associate immune cell signatures with survival outcomes following adjuvant treatment. In this study, the presence of CD103^+^ resident memory cells in the surgically resected tumor were associated with improved survival, regardless of treatment (62). Another study using samples from the SAKK16/14 trial of sequential neoadjuvant chemotherapy and perioperative immunotherapy performed tissue segmentation to identify the spatial distribution of intratumoral immune populations (63). This demonstrated that the degree of CD8 T-cell infiltration and the presence of tertiary lymphoid structures in pre-treatment biopsies were associated with improved EFS. In addition to identifying biomarkers in pre-treatment biopsies, some groups have attempted to use pathologic features to predict pathologic responses. One such group used deep learning approaches to develop a prediction model for pathologic response in NSCLC (64). This model was able to predict pathologic responses with AUC 0.848 and 0.831 for pCR and MPR, respectively. Importantly, the predictions from this model were also associated with survival outcomes in patients. Together, these studies demonstrate the feasibility and the prognostic potential of using digital pathology for biomarker discovery.

The breadth of clinical and translational research in early-stage NSCLC have identified numerous prognostic biomarkers that have helped accelerate discovery and raise the potential for more personalized care. The advances in computational power have led to the development of predictive models based on radiomic and pathologic data. However, these biomarkers largely remain prognostic rather than predictive, with no trials to date addressing adaptive treatment strategies based on these biomarkers. Even well-established prognostic biomarkers like pathologic response are limited, as most patients do not achieve a pCR. Better neoadjuvant strategies and more personalized escalation strategies are needed.

## 4 Ongoing trials and novel approaches

Several studies have explored novel combination approaches to neoadjuvant immunotherapy. Based on the utility of VEGF inhibition in the metastatic and refractory setting, the EAST ENERGY trial investigated the feasibility and efficacy of neoadjuvant pembrolizumab+ramicirumab in early-stage, PD-L1 positive NSCLC (65). This chemotherapy-free regimen was given for two cycles and yielded an MPR rate of 50% and pCR rate of 25%, with 22/24 (91.7%) of patients undergoing surgery. While further studies with larger sample sizes are needed, these findings raise the possibility that chemotherapy might be omitted in a subset of patients without compromising efficacy. Other approaches to escalate neoadjuvant therapy were explored in the NeoCOAST-2 platform trial (66). In this study, patients were randomized to durvalumab plus platinum-doublet chemotherapy or the CD73 inhibitor oleclumab, the NKG2A inhibitor monalizumab, or the TROP-2 antibody-drug conjugate datopotamab deruxtecan (dato-DXd). MPR and pCR rates were 41.9% and 20.3% for the oleclumab arm, 50.0% and 25.7% for the monalizumab arm, and 63.0% and 35.2% for the dato-DXd arm. While survival data are immature and randomized comparisons are needed, the robust increase in pathological response rates with dato-DXd suggests that using ADCs with PD-L1 inhibitors may represent a useful strategy in the neoadjuvant setting. Several studies are investigating the role of Trop-2 targeted ADCs in the neoadjuvant or adjuvant setting for NSCLC.

One intuitive approach based on the existing data is using perioperative immunotherapy in those patients who have negative prognostic features (e.g., a lack of a pCR, persistent ctDNA shedding). While perioperative trials such as Keynote 671, AEGEAN, and Checkmate 77T have been positive with improved pathologic responses, EFS, and OS, these trials randomized patients prior to the initiation of neoadjuvant therapy and did not include randomization to the adjuvant portion of therapy. As such, the relative contribution of the adjuvant immunotherapy remains unclear. Further, if a patient has less than a pCR, it is unclear whether continuing immunotherapy provides additional benefit. Data from trials have begun to address this question by evaluating outcomes across the spectrum of RVT. In CheckMate 816, there was a consistent decrement in survival with increasing RVT (29, 67).Similar findings were observed from the Keynote 671 trial, with worse EFS with higher RVT (68). However, in Keynote 671, the benefit of immunotherapy over chemotherapy was lost at high RVT, with the mEFS 15 months vs 18.5 months in the placebo arm (HR 0.90, 0.6-1.36). These findings suggest that RVT might help identify not only patients who do not require the adjuvant phase of immunotherapy but also those who might benefit from an escalated approach to their treatment.

One escalation strategy under investigation is providing additional stimulation to the immune system, including through the use of personalized antitumor vaccines. By generating an mRNA vaccine from peptides in the surgical resection specimen (mRNA-4157, V940), patient T-cells can be trained on a more refined panel of tumor antigens, theoretically providing more durable antitumor immunity. Phase I studies in resected NSCLC and melanoma showed durable responses with a reasonable safety profile (69). These durable responses and correlated increases in immunogenicity with V940 have led to a phase 3 study of V490+pembrolizumab for patients with NSCLC who did not achieve a pCR after neoadjuvant chemoimmunotherapy (70). If successful, this trial will provide a needed option for therapeutic escalation for patients with suboptimal responses after neoadjuvant therapy.

## 5 Conclusion

Advances in neoadjuvant and adjuvant immunotherapy have improved outcomes in early-stage NSCLC and have led to the approval of several chemoimmunotherapy regimens. From these studies, several prognostic biomarkers have been identified; however, escalation approaches for patients with suboptimal responses remain poorly defined. As additional trials accrue patients and as real-world experience increases, careful annotation of outcomes and biomarkers combined with novel computational approaches to link salient features can help realize the opportunity for truly personalized care in early-stage NSCLC.
